# Association Between Apelin and Atrial Fibrillation in Patients With High Risk of Ischemic Stroke

**DOI:** 10.3389/fcvm.2021.742601

**Published:** 2021-10-12

**Authors:** Allan Bohm, Peter Snopek, Lubomira Tothova, Branislav Bezak, Nikola Jajcay, Marianna Vachalcova, Tomas Uher, Marian Kurecko, Viera Kissova, Katarina Danova, Peter Olejnik, Peter Michalek, Tereza Hlavata, Katarina Petrikova, Viliam Mojto, Jan Kyselovic, Stefan Farsky

**Affiliations:** ^1^National Institute of Cardiovascular Diseases, Bratislava, Slovakia; ^2^3rd Department of Internal Medicine, Faculty of Medicine, Comenius University in Bratislava, University Hospital Bratislava, Bratislava, Slovakia; ^3^Premedix Academy, Bratislava, Slovakia; ^4^Department of Cardiology, Faculty Hospital Nitra, Nitra, Slovakia; ^5^St. Elizabeth University of Health and Social Work in Bratislava, Bratislava, Slovakia; ^6^Institute of Molecular Biomedicine, Faculty of Medicine, Comenius University, Bratislava, Slovakia; ^7^Faculty of Medicine, Comenius University, Bratislava, Slovakia; ^8^Faculty of Medicine, Slovak Medical University in Bratislava, Bratislava, Slovakia; ^9^Department of Complex Systems, Institute of Computer Science, Czech Academy of Sciences, Prague, Czechia; ^10^1st Department of Cardiology, East Slovak Institute of Cardiovascular Diseases, Košice, Slovakia; ^11^Faculty of Medicine, Pavol Jozef Safarik University, Košice, Slovakia; ^12^1st Department of Internal Medicine, Faculty of Medicine, Comenius University, Bratislava, Slovakia; ^13^Department of Laboratory Medicine, National Institute of Cardiovascular Diseases, Bratislava, Slovakia; ^14^House of the Heart (Dom Srdca), Slovak League Against Hypertension, Martin, Slovakia

**Keywords:** atrial fibrillation, apelin, biomarker, electrical atrial remodeling, ischemic stroke

## Abstract

**Background:** Atrial fibrillation (AF) is associated with high risk of stroke preventable by timely initiation of anticoagulation. Currently available screening tools based on ECG are not optimal due to inconvenience and high costs. Aim of this study was to study the diagnostic value of apelin for AF in patients with high risk of stroke.

**Methods:** We designed a multicenter, matched-cohort study. The population consisted of three study groups: a healthy control group (34 patients) and two matched groups of 60 patients with high risk of stroke (AF and non-AF group). Apelin levels were examined from peripheral blood.

**Results:** Apelin was significantly lower in AF group compared to non-AF group (*0.694* ± *0.148* vs. *0.975* ± *0.458* ng/ml, *p* = 0.001) and control group (*0.982* ± *0.060* ng/ml, *p* < 0.001), respectively. Receiver operating characteristic (ROC) analysis of apelin as a predictor of AF scored area under the curve (AUC) of *0.658*. Apelin's concentration of *0.969 [ng/ml]* had sensitivity = *0.966* and specificity = *0.467*. Logistic regression based on manual feature selection showed that only apelin and NT-proBNP were independent predictors of AF. Logistic regression based on selection from bivariate analysis showed that only apelin was an independent predictor of AF. A logistic regression model using repeated stratified K-Fold cross-validation strategy scored an AUC of 0.725 ± 0.131.

**Conclusions:** Our results suggest that apelin might be used to rule out AF in patients with high risk of stroke.

## Introduction

Atrial fibrillation (AF) is associated with high mortality, morbidity, and significant health care costs (1, 2). Despite substantial progress in cardiovascular prevention, constantly increasing incidence and prevalence of AF have reached dimensions of cardiovascular epidemic (3–5). As an independent factor, AF increases the risk of ischemic stroke 5-fold, as well as significantly contributes to the risk of heart failure and death (1, 6). Adequate anticoagulation therapy protects patients from these adverse events, but timely and accurate diagnosis remains a basic precondition (1, 7–11).

Currently available AF diagnostic tools are not sufficient. Standard 12-lead ECG is unreliable because of its low detection rate, especially in the setting of asymptomatic AF, and prolonged ECG monitoring is often impractical due to its high cost and inconvenience. Moreover, it is not always available due to high demand (12–14). There is an increasing need for a new, simple, cost-effective and accurate diagnostic tool, such as a biomarker detectable in peripheral blood.

Our knowledge of AF pathogenesis has evolved and emerging evidence strongly links AF with inflammation, oxidative stress and atrial fibrosis (15–20). Several plasmatic biomarkers for AF have been studied (21–25) and apelin, an endogenous regulatory peptide associated with many physiological and pathophysiological processes (26), has shown promising results (27–29). Among other effects on cardiovascular system, apelin shortens action potential duration in atrial myocytes via its effects on multiple ionic channels. It also affects the renin-angiotensin-aldosterone signaling pathway, acts as a second catalytic substrate for angiotensin-converting enzyme 2 (ACE2) and functions as an inotrope, all of which are processes directly or indirectly associated with AF (30, 31).

In our previous research that included only patients with low risk of stroke, we showed that apelin is significantly decreased in patients with AF compared to patients without AF (27, 28). Whether this result also applies to patients with cardiovascular comorbidities and high risk of stroke is unknown.

Our study sought to further investigate the relationship between apelin and atrial fibrillation and to determine apelin's predictive value for AF in patients with high risk of stroke.

## Materials and Methods

### Study Population

We designed a multicenter, matched-cohort study. Four Slovak hospitals in Bratislava, Malacky, Nitra and Kosice were included. The population consisted of three study groups: A healthy control group consisting of 34 patients without AF (control group) and two matched groups of 60 patients with high risk of stroke: one with atrial fibrillation (AF group) and the other without atrial fibrillation (non-AF group). The healthy control group consisted of random blood donors. Atrial fibrillation was excluded in both control and non-AF group based on the history and 12-lead ECG at the time of enrollment. The inclusion criteria for the AF group were: Age > 17 years, documented, non-valvular paroxysmal AF in the duration of more than 30 s (ECG documented), CHA2DS2-VASc score > 2 for males, CHA2DS2-VASc score > 3 for females and sinus rhythm at the time of inclusion. The inclusion criteria for the non-AF group were: Age > 17 years, CHA2DS2-VASc score > 2 for males, CHA2DS2-VASc score > 3 for females, sinus rhythm at the time of inclusion, no history of palpitations and 30 s AF exclusion using a continuous 7-day ECG Holter and additional 30-day ECG event recorder monitoring three times a day or when the patient felt unwell. Continuous 7-day ECG monitoring was performed using a QardioCore® device and 30-day ECG event recording was performed using a Hartmann Veroval®. The AF group and non-AF group were matched according to these parameters: age, gender, CHA2DS2-VASc parameters, left ventricular ejection fraction (LVEF): reduced (<40%), mid-range (40–49%) and preserved (≥50%), presence of diastolic dysfunction, glomerular filtration rate: (≥1.5 ml/s), (1.4–1 ml/s) and (0.9–0.5 ml/s), drugs (angiotensin-converting enzyme inhibitors and an angiotensin receptor blockers, betablockers, digoxin, amiodarone), body mass index (BMI): (<30 kg/m^2^), (30–39 kg/m^2^), and (≥40 kg/m^2^) and smoking (>5 cigarettes per day). Exclusion criteria for both groups were: electrical cardioversion <7 days prior to inclusion, acute coronary syndrome <1 month prior to inclusion, cardiac surgery <3 months prior to inclusion, acute or decompensated heart failure at the time of inclusion, pregnancy, cardiomyopathy, alcoholism (≥8 drinks/week), thyrotoxicosis, renal disease (dialysis/transplant/CrCl <0.5 ml/s), liver disease (cirrhosis/transaminase > 3x ULN/bilirubin > 2x ULN), mechanical prosthetic valve, severe mitral stenosis, class I and IV antiarrhythmic drugs usage in the last month, class III antiarrhythmic drugs usage in the last 3 months.

The study was approved by the Ethics Committee of the National Cardiovascular Institute, Bratislava, Slovakia and a written informed consent was obtained from all patients and donors in the control group.

### Data Collection and Biochemical Analysis

In AF and non-AF groups, baseline clinical data were obtained during ambulatory visits or during a hospitalization and were recorded into an electronic online case report form. Peripheral fasting blood was taken in the morning using K3EDTA tubes. In the control group, baseline clinical data and fasting blood samples were collected at the time of blood donation. The blood was centrifuged at 2,700 *g* for 5 min and the obtained plasma samples were stored at −80°C. The apelin-12 concentration was measured using a commercially available ELISA kit (Phoenix Pharmaceutical, Karlsruhe, Germany) in plasma samples. Fifty microliters of plasma samples were used for measurement according to the manufacturer's protocol.

### Statistical Methods

Continuous variables are presented as sample means and standard deviations. Normality of data was tested using a Shapiro–Wilk test and inspected on Q-Q plots, with homoscedasticity assessed using Levene's test. Classic or Welch ANOVA was employed to analyze the between group differences based on equality of variances, followed by *post-hoc* tests (Tukey-HSD or Games-Howell, respectively) in order to study pairwise differences between groups. Between group differences for categorical variables were estimated using the χ^2^ test of independence with λ = −*2* (Neyman test). All correlations were computed using Spearman's correlation coefficient in order to suppress the effect of tentative outliers. All logistic regression models were fitted either in *sklearn* (with *Elastic-Net* regularization with equal L1 and L2 ratios, and *saga* solver) or *statsmodels* (with the iteratively reweighted least squares method) python libraries, and all receiver operating characteristic (ROC) curves and area under the curve (AUC) statistics were computed using the *sklearn* python library. Before entering the logistic regression, all data were scaled using the standard scaler (to zero mean and unit variance). *P*-values < 0.05 were considered statistically significant.

Based upon our previous research, the expected mean difference in apelin concentration was 0.15 ng/ml with a standard deviation of 0.14. Assuming an alpha of 0.05 and 90% power, the minimum sample size was 24 patients in each matched group.

Data were analyzed using Python version 3.7.9 (https://www.python.org/) with appropriate libraries (for statistical analyses *pingouin* package version 0.3.8: https://pingouin-stats.org/, for regression models and their statistics *statsmodels* package version 0.12.1: https://www.statsmodels.org/, *scikit-learn* package version 0.23.2: https://scikit-learn.org/, and *RStudio* 1.2.5033 (32) which was also used for sample size calculation.

## Results

### Baseline Characteristics

A total of 94 patients were enrolled in the study: 30 in the AF group, 30 in the non-AF group and 34 in the healthy control group. Patient characteristics are presented in Tables 1, 2A–C. There were statistically significant differences between the AF and non-AF groups in CRP levels [4.94 ± 5.31 vs. 7.62 ± 25.83 (mg/l), respectively, *p* = 0.012], NT-proBNP levels (664.82 ± 773.48 vs. 286.84 ± 297.27, respectively, *p* = 0.026), Apelin levels (0.69 ± 0.15 vs. 0.98 ± 0.45, respectively, *p* = 0.032) and antithrombotic therapy (see Tables 1, 2A,B). Patients in the control group were significantly younger than patients in the AF and non-AF groups.

**Table 1:** Baseline demographics of the study population.

	**Non-AF group** **(*n* = 30)**	**AF group** **(*n* = 30)**	***p*** **-value**
Age (years)	71.83 ± 8.00	73.63 ± 7.40	0.378
Male gender (%)	19 (63.3%)	19 (63.3%)	>0.999
Weight (kg)	83.93 ± 12.20	82.63 ± 15.89	0.728
Height (cm)	170.57 ± 9.04	171.10 ± 9.68	0.829
BMI (kg/m^2^)	28.86 ± 3.53	28.10 ± 4.09	0.45
Smoking (>5 cigarettes per day) (%)	2 (6.7%)	1 (3.3%)	>0.999
Systolic blood pressure (mmHg)	134.13 ± 12.48	131.37 ± 9.77	0.366
Diastolic blood pressure (mmHg)	79.13 ± 7.23	75.70 ± 8.36	0.214

*Data in the table are presented as mean ± standard deviation or n (%).*

*AF, atrial fibrillation; BMI, body mass index*.

**Table 2A:** Patient characteristics: echocardiography and laboratory parameters.

	**Non-AF group** **(*n* = 30)**	**AF group** **(*n* = 30)**	***p*** **-value**
**Echocardiography**			
Left ventricular end-diastolic diameter (mm)	48.73 ± 4.73	48.80 ± 5.34	0.96
Diameter of left atrium in PLAX (Parasternal long axis) (mm)	42.87 ± 5.16	43.00 ± 5.12	0.922
Diastolic dysfunction	0.93 ± 0.73	1.17 ± 0.73	0.295
Left ventricular hypertrophy (%)	13 (43.3%)	14 (46.7%)	>0.999
**Laboratory parameters**			
D-dimer (ug/ml)	314.22 ± 391.99	308.33 ± 443.38	>0.999
Fibrinogen (g/l)	3.71 ± 1.24	3.56 ± 0.58	0.605
CRP (mg/l)	7.62 ± 25.83	4.94 ± 5.31	0.012
NT-proBNP (ng/l)	286.84 ± 297.27	664.82 ± 773.48	0.026
Hs-troponin (ng/l)	11.55 ± 6.77	36.02 ± 96.97	0.071
Apelin (ng/ml)	0.98 ± 0.45	0.69 ± 0.15	0.032
Creatinine (umol/l)	82.00 ± 15.80	85.42 ± 16.53	0.425
Creatinine clearance (ml/s)	1.25 ± 0.21	1.20 ± 0.22	0.376

*Data in the table are presented as mean ± standard deviation or n (%).*

*AF, atrial fibrillation; CRP, C-reactive protein; Hs-troponin, High-sensitivity troponin; NT-proBNP, N-terminal fragment of brain natriuretic peptide*.

**Table 2B:** Patient characteristics: medical history and medication.

	**Non-AF group** **(*n* = 30)**	**AF group** **(*n* = 30)**	***p*** **-value**
**Medical history**			
AF burden (months)	0	29.85 ± 28.43	N/A
Ischemic stroke/TIA	1.14 ± 0.35	1.25 ± 0.43	0.677
STEMI	1.20 ± 0.40	1.00 ± 0.00	0.606
NSTEMI	1.33 ± 0.47	1.33 ± 0.47	0.792
Ventricular tachycardia/ventricular fibrillation (%)	1 (3.3%)	2 (6.7%)	>0.999
Arterial hypertension (%)	29 (96.7%)	29 (96.7%)	>0.999
Pulmonary embolism (%)	0 (0.0%)	1 (3.3%)	>0.999
Deep vein thrombosis (%)	0 (0.0%)	3 (10.0%)	0.116
Peripheral arterial disease/aortic plaque (%)	10 (33.3%)	13 (43.3%)	0.594
Left ventricular hypertrophy (%)	13 (43.3%)	14 (46.7%)	>0.999
Stable coronary artery disease (%)	8 (26.7%)	4 (13.3%)	0.32
Chronic obstructive pulmonary disease (COPD) (%)	1 (3.3%)	5 (16.7%)	0.141
Obstructive sleep apnea (OSA) (%)	1 (3.3%)	0 (0.0%)	>0.999
Severe valvulopathy (%)	0 (0.0%)	0 (0.0%)	>0.999
Electrical cardioversion (%)	0 (0.0%)	2 (6.7%)	0.408
Pharmacological cardioversion (%)	0 (0.0%)	3 (10.0%)	0.116
CHADS2-VASc	3.7	3.7	N/A
**Medication**			
ACE- inhibitor/ARB (%)	28 (93.3%)	26 (86.7%)	0.663
Spironolactone/Eplerenone (%)	1 (3.3%)	1 (3.3%)	>0.999
Beta-blocker (%)	26 (86.7%)	26 (86.7%)	>0.999
Digoxin (%)	1 (3.3%)	1 (3.3%)	>0.999
Proton pump inhibitors (%)	6 (20.0%)	13 (43.3%)	0.083
Antidepressants/Antipsychotics (%)	0 (0.0%)	2 (6.7%)	0.408
Acetylsalicylic acid (%)	16 (53.3%)	8 (26.7%)	0.056
Clopidogrel (%)	7 (23.3%)	3 (10.0%)	0.279
Prasugrel (%)	1 (3.3%)	0 (0.0%)	>0.999
Ticagrelor (%)	0 (0.0%)	0 (0.0%)	>0.999
Warfarin (%)	1 (3.3%)	9 (30.0%)	0.001
Dabigatran etexilat (%)	0 (0.0%)	4 (13.3%)	0.021
Rivaroxaban (%)	0 (0.0%)	0 (0.0%)	>0.999
Apixaban (%)	0 (0.0%)	8 (26.7%)	0.001
Edoxaban (%)	0 (0.0%)	1 (3.3%)	>0.999

*Data in the table are presented as mean ± standard deviation or n (%).*

*ACE, Angiotensin-converting enzyme; ARB, angiotensin receptor blocker; NSTEMI, Non-ST-elevation myocardial infarction; STEMI, ST-Elevation myocardial infarction; TIA, transient ischemic attack*.

**Table 2C:** Patient characteristics: atrial fibrillation patients vs. non-atrial fibrillation patients vs. control group.

**Characteristics**	**Non-AF group** **(*n* = 30)**	**AF group** **(*n* = 30)**	**Control group** **(*n* = 34)**	***p*** **-value**
Age (years)	71.83 ± 8.00	73.63 ± 7.40	41.03 ± 9.34	<0.001
Apelin (ng/ml)	0.98 ± 0.45	0.69 ± 0.15	0.98 ± 0.06	= 0.001
Male gender (%)	19 (63.3%)	19 (63.3%)	13 (38.2%)	0.0544

*Data in the table are presented as mean ± standard deviation or n (%).*

*AF, atrial fibrillation*.

**Figure 1:**
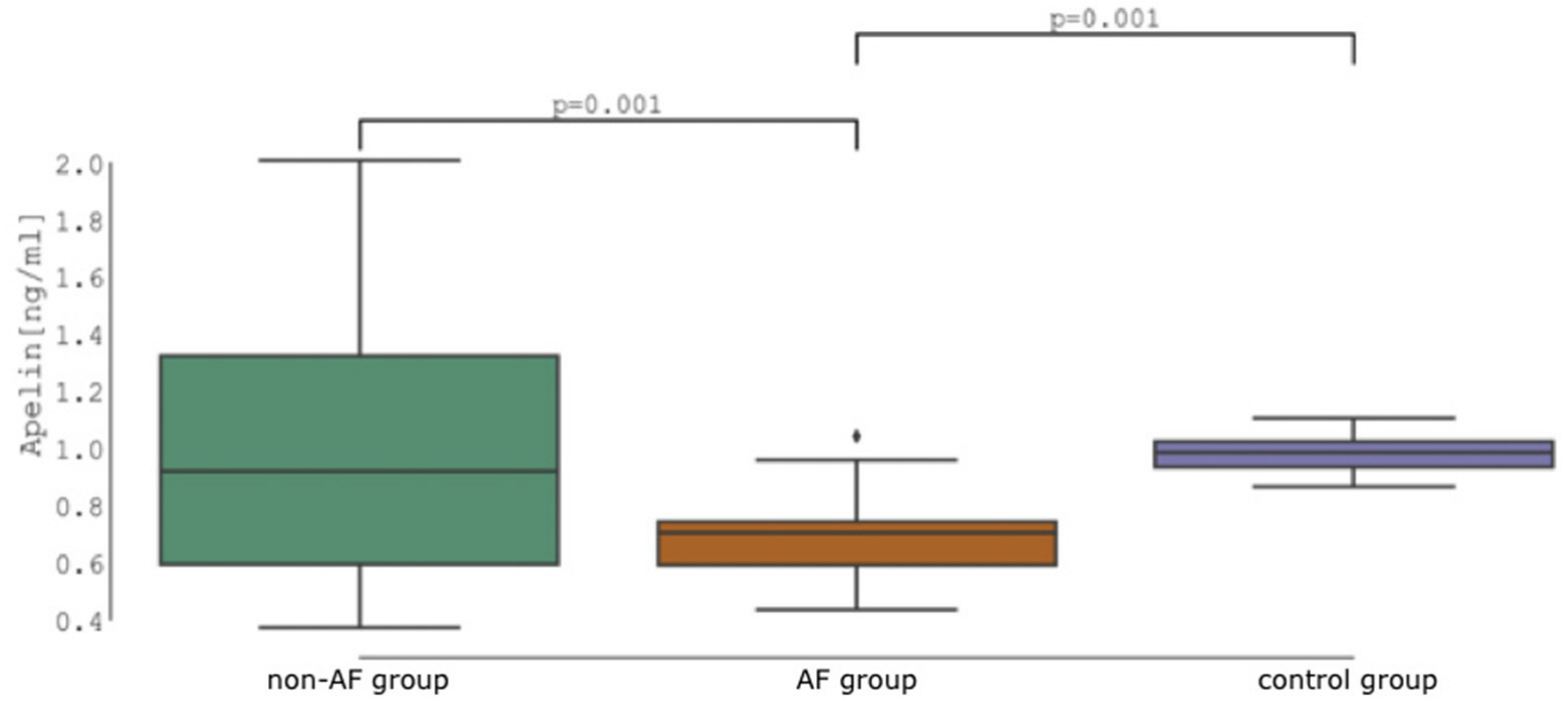
Apelin concentration: Non-atrial fibrillation patients (non-AF group) vs. atrial fibrillation patients (AF group) vs. control group.

The analysis of variance test (ANOVA) for all three groups showed a significant group effect on apelin concentrations with *F*_(2, 90)_ = 10.67, *p* < 0.001, $\eta_{p}^{2}$ = 0.192 with statistical power *0.994* given our number of participants. Subsequent analysis showed significant difference in apelin concentration between healthy controls and patients with AF (*0.982* ± *0.060* vs. *0.694* ± *0.148* ng/ml, *p* = 0.001, *d* = 1.044) as well as between patients with and without AF (*0.694* ± *0.148* vs. *0.975* ± *0.458* ng/ml, *p* = 0.001, *d* = −1.021), respectively. The difference between healthy controls and patients without AF was not significant (*0.982* ± *0.060* vs. *0.975* ± *0.458* ng/ml, *p* = 0.900, *d* = 0.023) (Figure 1).

**Figure 2:**
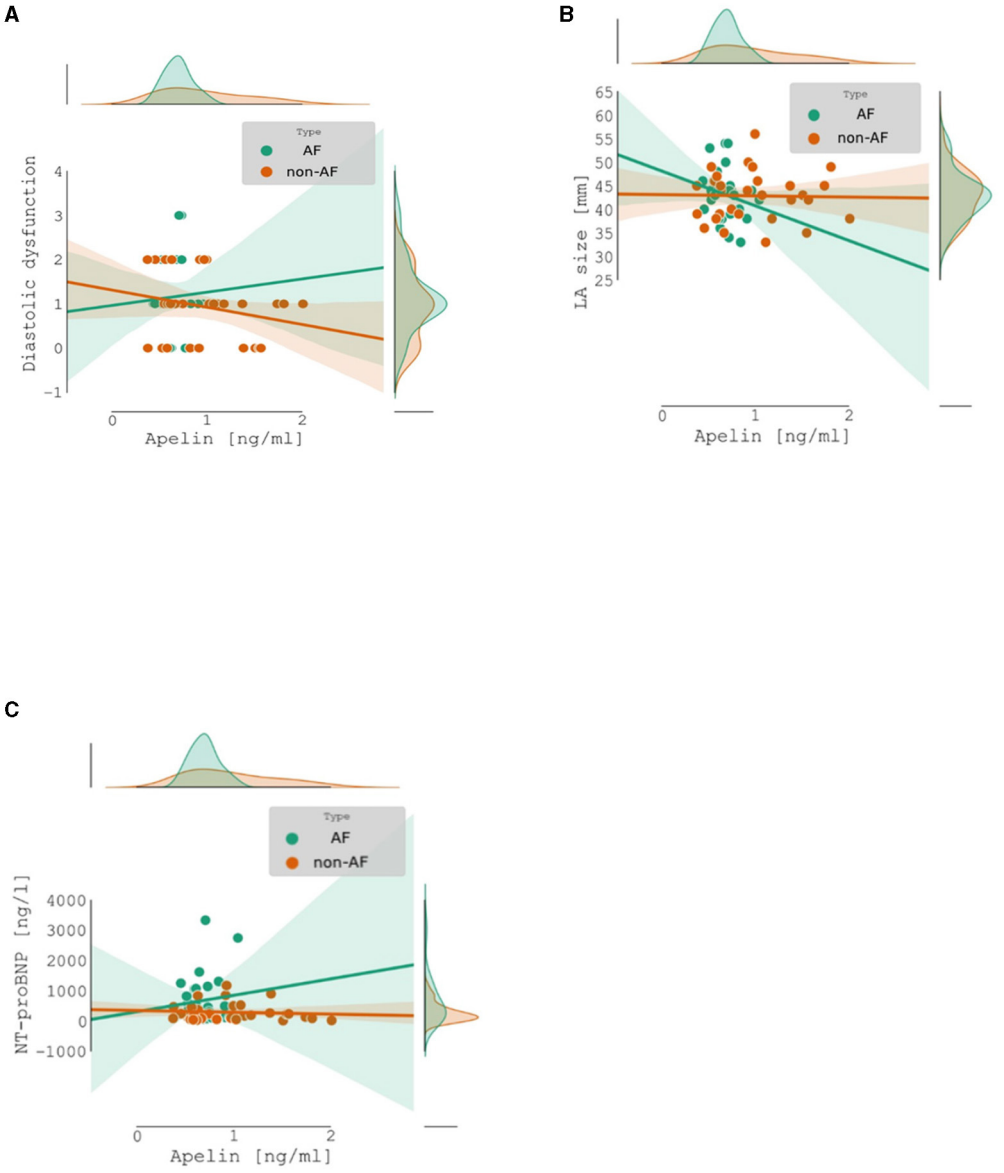
Apelin concentration: Non-atrial fibrillation patients (no-AF) vs. atrial fibrillation patients (AF): **(A)** correlation between apelin concentration and diastolic dysfunction; **(B)** correlation between apelin concentration and left atrium diameter; **(C)** correlation between apelin concentration and NT-proBNP.

There was no significant correlation between apelin concentration and diastolic dysfunction [*Spearman's r* = −*0.126, CI 95% (*−*0.37, 0.13), p* = 0.341], left atrium diameter in parasternal short axis [mm] [*Spearman's r* = −*0.097, CI 95% (*−*0.34, 0.16), p* = 0.466], and NT-proBNP [ng/l] [*Spearman's r* = −*0.147, CI 95% (*−*0.39, 0.11), p* = 0.267] (Figure 2).

**Figure 3:**
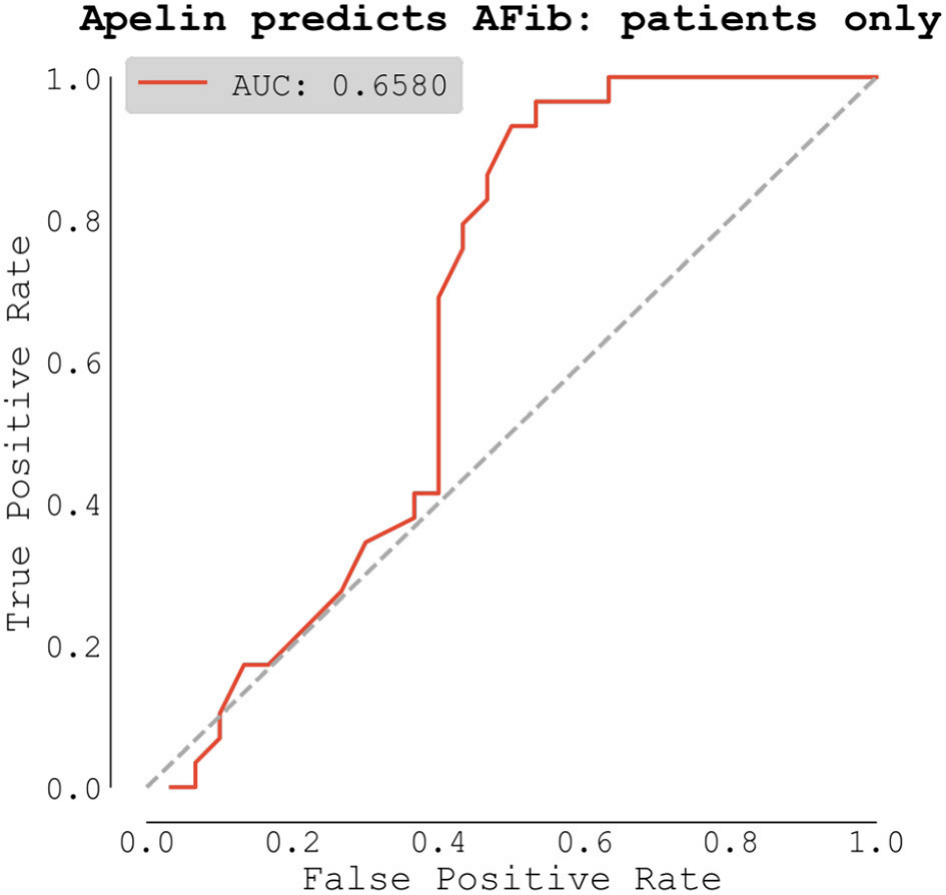
Receiver operating characteristic (ROC) analysis of apelin as a predictor of atrial fibrillation (AF).

ROC analysis of apelin as a predictor of AF scored AUC = 0.658. T = 0.658. The ideal threshold of *apelin concentration was 0.969 [ng/ml] with* accuracy of *0.712*, sensitivity of *0.966*, and specificity of *0.467*, respectively (Figure 3).

**Figure 4:**
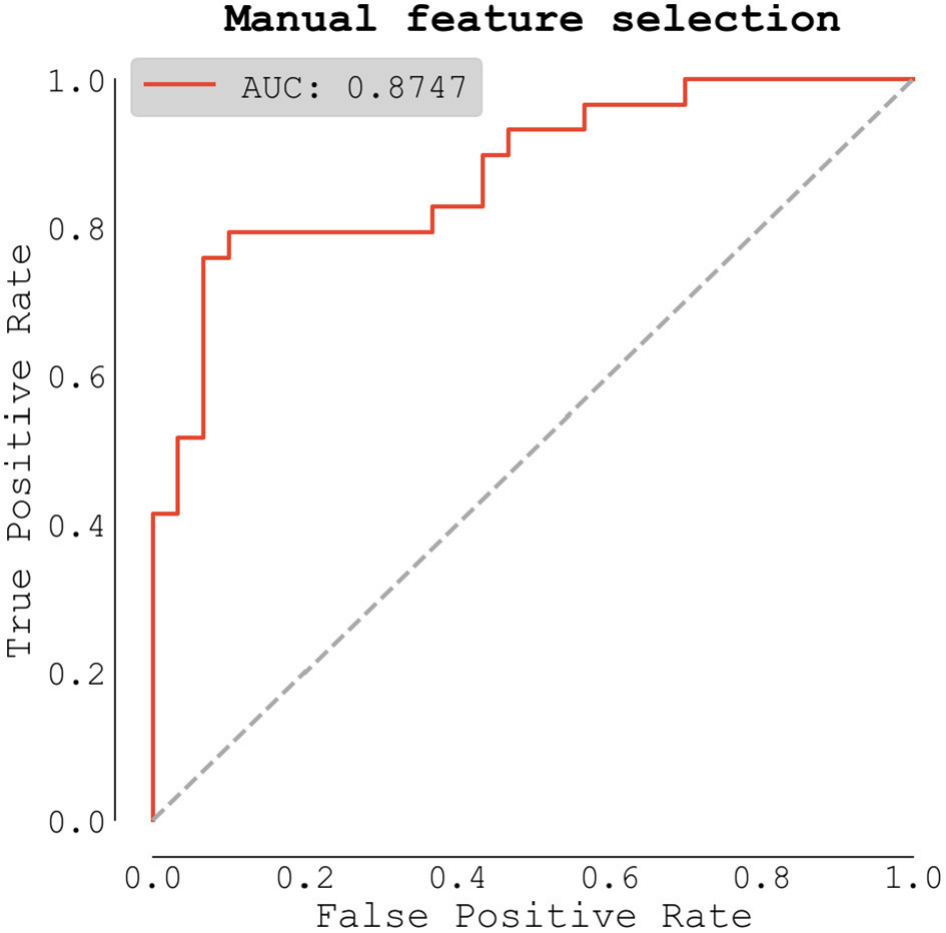
Logistic regression model (all patient data).

Finally, we built a logistic regression model for classifying AF using multiple predictors, including apelin. We compared two approaches to this problem, with the first being the manual feature selection based on known predictors of AF from available literature. We selected 16 predictors from our gathered data and fitted a logistic regression model using our patients' data. The model trained on all data scored *AUC* = *0.875* (Figure 4).

The full list of predictors with their coefficients and *p*-values can be seen in Table 3A. Only two predictors were statistically significant with *p*-values lower than 0.05: *apelin*, and *NT-proBNP*.

**Table 3A:** Logistic regression model for AF predictors.

**Predictor**	**Coef**	**(95% CI)**	***p*** **-value**
(Intercept)	−2.875	(−5.958 to 0.209)	0.068
Signs of heart failure (%)	−0.606	(−2.856 to 1.645)	0.598
Diastolic dysfunction (Grade)	1.512	(−0.622 to 3.647)	0.165
Chronic obstructive pulmonary disease (COPD) (%)	3.745	(0.005 to 7.485)	0.05
Vascular disease (%)	−0.175	(−2.005 to 1.656)	0.852
Gender (%)	1.64	(−0.495 to 3.775)	0.132
Diabetes Mellitus (%)	1.252	(−1.073 to 3.577)	0.291
D-Dimer (ug/ml)	−0.325	(−1.19 to 0.541)	0.462
Systolic blood pressure (mmHg)	0.263	(−0.701 to 1.228)	0.593
Age (years)	−0.47	(−1.398 to 0.457)	0.320
NT-proBNP (ng/l)	1.823	(0.251 to 3.396)	0.023
Diastolic blood pressure (mmHg)	−0.45	(−1.447 to 0.548)	0.377
BMI (kg/m^2^)	0.047	(−0.916 to 1.011)	0.923
Apelin (ng/ml)	−1.936	(−3.551 to −0.320)	0.019
CRP (mg/l)	−0.222	(−1.068 to 0.624)	0.607
Creatinine (umol/l)	0.464	(−0.605 to 1.534)	0.395
Diameter of left atrium in PLAX (Parasternal long axis) (mm)	−0.645	(−1.81 to 0.520)	0.278

*Data in the table are presented as mean ± standard deviation or n (%).*

*AF, atrial fibrillation; BMI, body mass index; CRP, C-reactive protein; NT-proBNP, N-terminal fragment of brain natriuretic peptide*.

**Figure 5:**
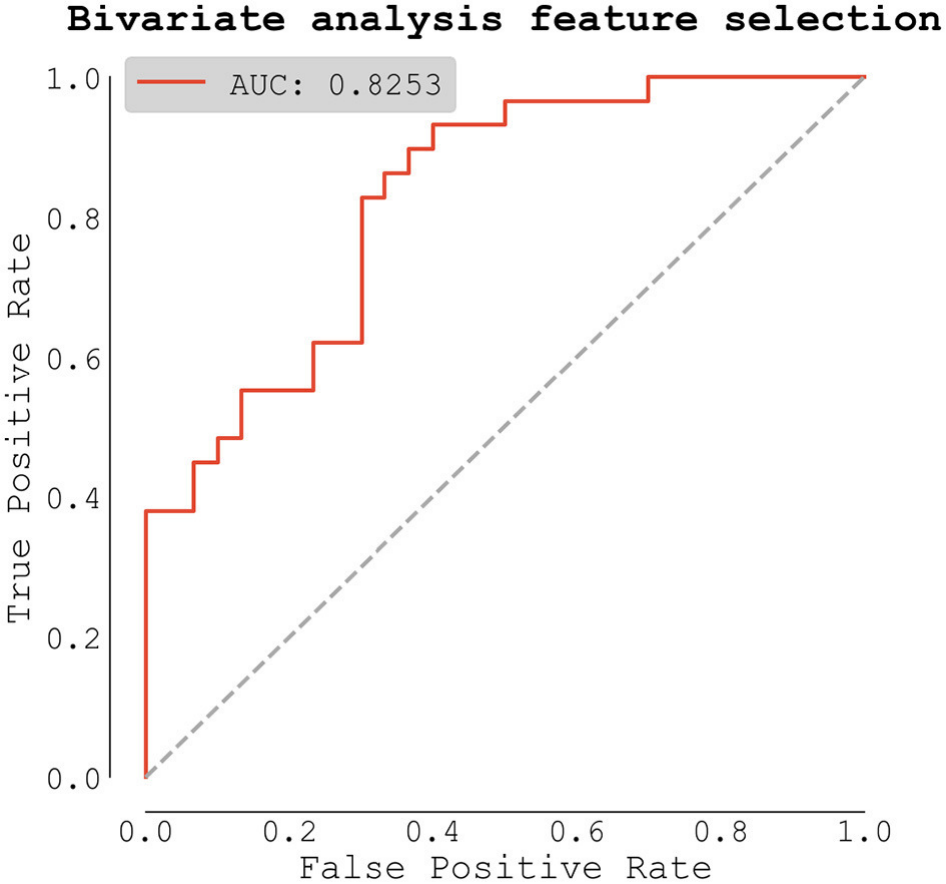
Logistic regression model based on selection from bivariate analysis (predictors with *p* < 0.1).

The second, data-driven route was to compute bivariate analysis (significant differences in our dataset between AF and no AF patients) and include all predictors, whose differences between groups had *p*-value lower than 0.1 (based on *t*-test, Mann-Whitney *U*-test, or χ^2^ test where appropriate). Differences in medication were not included in this analysis because they directly depend on the presence of AF. This landed us with four predictors (of course, including apelin) and the final model scored *AUC* = *0.825* (Figure 5). In this model, only apelin scored *p*-value lower than preselected threshold of 0.05. The full list of predictors with their coefficients and *p*-values can be seen in Table 3B.

**Table 3B:** Logistic regression model for AF predictors based on selection from bivariate analysis (predictors with *p* < 0.1).

**Predictor**	**Coef**	**(95% CI)**	***p*** **-value**
(Intercept)	0.409	(−0.653 to 1.471)	0.450
Apelin (ng/ml)	−1.019	(−1.915 to −0.123)	0.026
Hs-troponin (ng/l)	3.907	(−1.512 to 9.327)	0.158
NT-proBNP (ng/l)	0.777	(−0.142 to 1.696)	0.097
CRP (mg/l)	−0.321	(−1.061 to 0.418)	0.395

*Data in the table are presented as mean ± standard deviation or n (%).*

*AF, atrial fibrillation; CRP, C-reactive protein; Hs-troponin, High-sensitivity troponin; NT-proBNP, N-terminal fragment of brain natriuretic peptide*.

**Figure 6:**
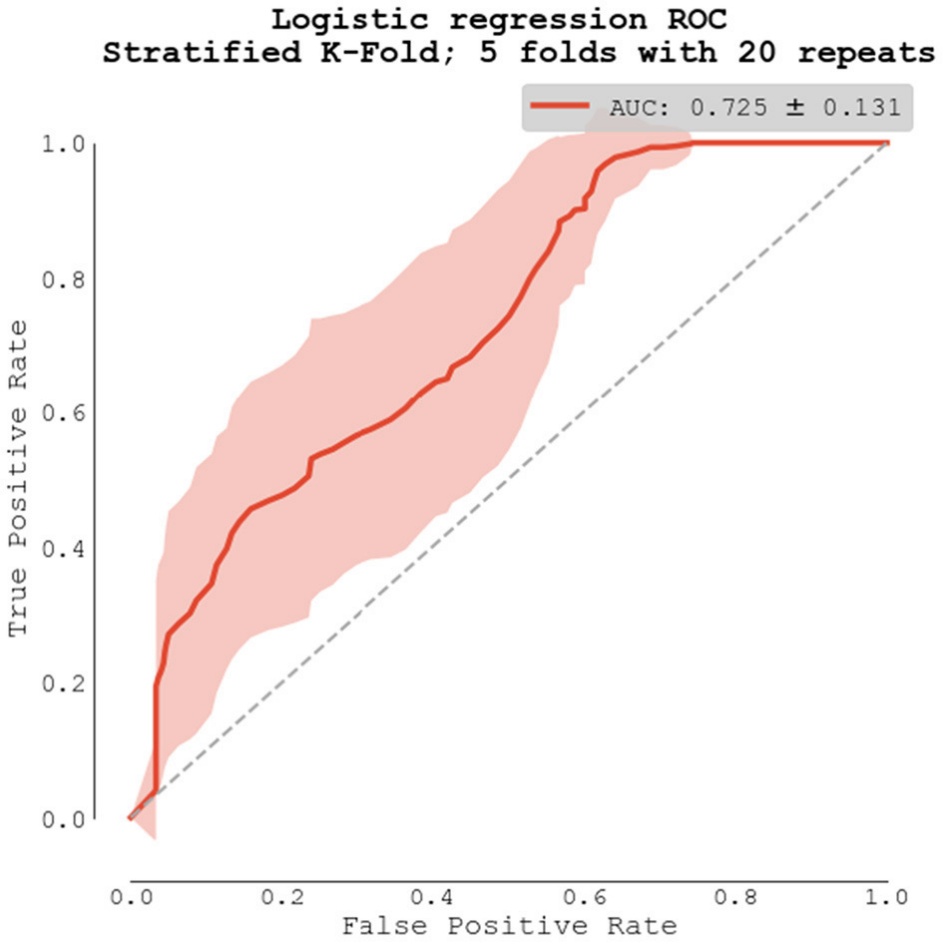
Logistic regression model using repeated stratified K-Fold cross-validation strategy.

To assess the true model performance, we selected predictors from our bivariate analysis with *p*-value < 0.1, and repeatedly trained logistic regression model using repeated stratified K-Fold cross-validation strategy. The receiver operating characteristic (ROC) was computed only from testing dataset. Our final model scored *AUC* = *0.725* ± *0.131, with improved sensitivity: 0.851* ± *0.209 and specificity: 0.685* ± *0.250*. Full ROC curve showed as mean ± one standard deviation can be seen in Figure 6.

## Discussion

Our study demonstrated that in matched cohorts of patients with cardiovascular comorbidities and high risk of stroke, the cohort with AF had significantly lower concentration of apelin compared to the cohort without AF. Similar, there was a statistically significant difference in apelin concentration between patients with AF and the healthy control group.

Further analysis of known AF contributors (1, 32) in our dataset demonstrated that only apelin and NT-proBNP were independent predictors of AF. Increased levels of NT-proBNP in patients suffering from AF have been observed in several studies and their association is well-established (33–35). There are several unmeasured factors such as amount of exercise (36, 37) or dietary intake (38–40) which may alter apelin plasmatic levels. These changes may be pronounced between patient and healthy control group, however, should not be significant between matched cohorts. Additionally, it is not possible to completely eliminate the potential influence of medication on plasmatic levels of apelin. However, there was no statistically significant difference between plasmatic levels of apelin when comparing high risk patients in non-AF group and healthy donors receiving no chronic medication.

Based on ROC analysis, apelin was able to predict AF with an AUC of 66%. By setting the apelin level threshold to 0.969 [ng/ml] with the aim of maximizing sensitivity we demonstrated a classification accuracy of 0.712, sensitivity of 0.966, and specificity of 0.467.

Our previous research confirmed that apelin has high sensitivity and specificity to predict and quantify AF in patients with minimal cardiovascular comorbidities and low risk of stroke (27, 28). However, this result is not sufficient for use in clinical practice where there would be more complex cases and it would not be known whether apelin would be able to provide sufficient diagnostic power in patients with multiple comorbidities who are at high risk of stroke. Furthermore, young patients with a low risk profile and lone AF *(reflected in low CHA2DS2-VASc score)* do not meet existing criteria for anticoagulation treatment. To address these questions, we designed a study where apelin was studied in a high-risk cohort of patients with AF and multiple cardiovascular comorbidities. Patients with persistent/permanent AF were not included in this study because we wanted to study if apelin is reduced in the setting of paroxysmal AF and therefore if it potentially can be used for AF detection (e.g., in patients after cryptogenic stroke with no symptoms of arrhythmia).

We were able to confirm good sensitivity, however specificity for AF was low. The APJ receptor for apelin is detectable in many central and peripheral tissues (41, 42) and compelling evidence demonstrates that this complex is involved in a large number of physiological and pathophysiological processes (43, 44). Specificity for AF is therefore limited in patients with comorbidities. This situation could be overcome by including variables besides apelin in a classification model for AF detection.

The Stratified K-Fold cross-validation strategy was performed to ascertain the performance of logistic regression models and to compare this result with the performance of apelin alone. The overall predictive value increased from 66 to 73*% with improved sensitivity: 0.851* ± *0.209 and specificity: 0.685* ± *0.250*. These results suggest a potential improvement in the predictive value of apelin when incorporate into a multi-factor scoring system. The potential benefit of multi-factorial biomarker-based prediction models has already been described in several studies (35, 45, 46).

Optimal patient selection could improve the predictive value of apelin or apelin-based scoring systems for AF. For example, in the case of heart failure with decreased LV ejection fraction, some studies have reported decreased, unaltered or even increased plasma levels compared to control subjects (47, 48). Therefore, patients with reduced LVEF were excluded from our study. On the other hand, patients with heart failure with preserved ejection fraction (HFpEF) were included in our study and they did not show any association with apelin levels. Our observations suggest that apelin, despite its low specificity in the presence of several comorbidities indicating high risk of stroke, could still be used to rule out AF due to its high sensitivity. This, however, should be validated further in a larger cohort of patients.

We also hypothesized about the potential cause of apelin reduction in AF. Previous studies including our findings (28, 49) showed that increased stretch might play a pathophysiological role in decreased apelin concentration. However, in the present study, apelin showed no statistically significant correlation with left atrium (LA) size, NT-proBNP and diastolic dysfunction, all of which are known and verified risk factors for AF development and continuation, and reflect elevated pressure and volume in the atrium. In the context of our present findings, we hypothesize that these previously reported correlations were not causal and that apelin more likely reflects electrical remodeling rather than structural remodeling. This theory also corresponds with experimental findings showing that apelin increases atrial conduction velocity, refractoriness, shortens action potential, affects multiple ionic currents and prevents the inducibility of atrial fibrillation (50, 51).

We believe that our results encourage further research of apelin as a biomarker that might be used to rule out atrial fibrillation.

### Study Limitations

Our study had several limitations. The predictive value of apelin with multiple risk factors model was not validated on an independent cohort. However, the cross-validation strategy using repeated K-Fold was used to substitute the independent cohort validation. Secondly, although our inclusion criteria were relatively broad, there are still many unmeasured factors that could alter apelin plasmatic levels. Thirdly, apelin levels may change during the natural history of atrial fibrillation and our study did not follow changes of apelin levels over time. Lastly, a matched-cohort design cannot assess a causal relationship between apelin and AF. Thus, while our results are provocative, they need to be confirmed in future studies.

## Conclusion

Our results showed that low level of apelin has good sensitivity for atrial fibrillation even in the setting of multiple cardiovascular comorbidities that increase the risk of ischemic stroke. Additional research is needed to verify whether apelin could be used in clinical practice to rule out atrial fibrillation and to improve AF screening in patients with increased risk of ischemic stroke.

## Data Availability Statement

The original contributions presented in the study are included in the article/Supplementary Material, further inquiries can be directed to the corresponding author.

## Ethics Statement

The studies involving human participants were reviewed and approved by Ethics Committee of the National Cardiovascular Institute, Bratislava, Slovakia. The patients/participants provided their written informed consent to participate in this study.

## Author Contributions

AB conceived the ideas and designed the study. AB, PS, MV, MK, and TU conducted the study. AB, BB, and NJ analyzed the data. LT performed biochemical analyses. AB, BB, NJ, TH, and KP wrote the manuscript. SF, JK, VM, VK, PM, KD, and PO provided supervision. All the authors have read and approved the final version for publication.

## Funding

This work was supported by independent research grants provided by the Ministry of Education, Science, Research and Sport of the Slovak Republic VEGA [Grant Number 1/0563/21] and by Pfizer. There was no involvement of grantors in the collection, analysis and interpretation of data, writing of the manuscript, and in the decision to submit the article for publication.

## Conflict of Interest

The authors declare that the research was conducted in the absence of any commercial or financial relationships that could be construed as a potential conflict of interest.

## Publisher's Note

All claims expressed in this article are solely those of the authors and do not necessarily represent those of their affiliated organizations, or those of the publisher, the editors and the reviewers. Any product that may be evaluated in this article, or claim that may be made by its manufacturer, is not guaranteed or endorsed by the publisher.

## References

[1] Hindricks G Potpara T Dagres N Arbelo E Bax JJ Blomström-Lundqvist C. 2020 ESC guidelines for the diagnosis and management of atrial fibrillation developed in collaboration with the European association for cardio-thoracic surgery (EACTS): the task force for the diagnosis and management of atrial fibrillation of the European society of cardiology (ESC) developed with the special contribution of the European heart rhythm association (EHRA) of the ESC. Eur Heart J. (2021) 42:373–498. 10.1093/eurheartj/ehaa612 32860505

[2] Vermond RA Geelhoed B Verweij N Tieleman RG Van der Harst P Hillege HL. Incidence of atrial fibrillation and relationship with cardiovascular events, heart failure, and mortality: a community-based study from the Netherlands. J Am Coll Cardiol. (2015) 66:1000–7. 10.1016/j.jacc.2015.06.1314 26314526

[3] Chamberlain AM Brown RD Jr Alonso A Gersh BJ Killian JM Weston SA. No decline in the risk of stroke following incident atrial fibrillation since 2000 in the community: a concerning trend. J Am Heart Assoc. (2016) 5:e003408. 10.1161/JAHA.116.003408 27412902 PMC4937280

[4] Lippi G Sanchis-Gomar F Cervellin G. Global epidemiology of atrial fibrillation: an increasing epidemic and public health challenge. Int J Stroke. (2021) 16:217–21. 10.1177/1747493019897870 31955707

[5] Wilhelm M. How to best prevent cardioembolic stroke? Eur J Prev Cardiol. (2020) 26:961–63. 10.1177/2047487319842250 30971129

[6] Song Z Xu K Hu X Jiang W Wu S Qin M. A study of cardiogenic stroke risk in non-valvular atrial fibrillation patients. Front Cardiovasc Med. (2020) 7:604795. 10.3389/fcvm.2020.604795 33244472 PMC7683797

[7] Saglietto A Gaita F De Ponti R De Ferrari GM Anselmino M. Catheter ablation vs. Anti-arrhythmic drugs as first-line treatment in symptomatic paroxysmal atrial fibrillation: a systematic review and meta-analysis of randomized clinical trials. Front Cardiovasc Med. (2021) 8:664647. 10.3389/fcvm.2021.664647 34095254 PMC8175669

[8] Asad ZUA Yousif A Khan MS Al-Khatib SM Stavrakis S. Catheter ablation versus medical therapy for atrial fibrillation: a systematic review and meta-analysis of randomized controlled trials. Circ Arrhythm Electrophysiol. (2019) 12:e007414. 10.1161/CIRCEP.119.007414 31431051

[9] Barnett AS Kim S Fonarow GC Thomas LE Reiffel JA Allen LA. Treatment of atrial fibrillation and concordance with the American heart association/American college of cardiology/heart rhythm society guidelines: findings from ORBIT-AF (outcomes registry for better informed treatment of atrial fibrillation). Circ Arrhythm Electrophysiol. (2017) 10:e005051. 10.1161/CIRCEP.117.005051 29141842

[10] Taggar JS Coleman T. Screening for atrial fibrillation in primary care: from recommendation to implementation. Eur J Prev Cardiol. (2016) 23:1880–2. 10.1177/2047487316652976 27256828

[11] Kovacs RJ Flaker GC Saxonhouse SJ Doherty JU Birtcher KK Cuker A. Practical management of anticoagulation in patients with atrial fibrillation. J Am Coll Cardiol. (2015) 65:1340–60. 10.1016/j.jacc.2015.01.049 25835447

[12] Murali S Brugger N Rincon F Mashru M Cook S Goy JJ. Cardiac ambulatory monitoring: new wireless device validated against conventional holter monitoring in a case series. Front Cardiovasc Med. (2020) 7:587945. 10.3389/fcvm.2020.587945 33330650 PMC7733961

[13] Young B. New standards for ECG equipment. J Electrocardiol. (2019) 57:S1–S4. 10.1016/j.jelectrocard.2019.07.013 31387696

[14] Freedman B Camm J Calkins H Healey JS Rosenqvist M Wang J. Screening for atrial fibrillation: a report of the AF-screen international collaboration. Circulation. (2017) 135:1851–67. 10.1161/CIRCULATIONAHA.116.026693 28483832

[15] Segers VFM De Keulenaer GW. Autocrine signaling in cardiac remodeling: a rich source of therapeutic targets. J Am Heart Assoc. (2021) 10:e019169. 10.1161/JAHA.120.019169 33470124 PMC7955414

[16] Xiao Z Reddy DPK Xue C Liu X Chen X Li J. Profiling of miR-205/P4Ha3 following angiotensin II-induced atrial fibrosis: implications for atrial fibrillation. Front Cardiovasc Med. (2021) 8:609300. 10.3389/fcvm.2021.609300 33981730 PMC8107220

[17] Deng Y Liu F Yang X Xia Y. The key role of uric acid in oxidative stress, inflammation, fibrosis, apoptosis, and immunity in the pathogenesis of atrial fibrillation. Front Cardiovasc Med. (2021) 8:641136. 10.3389/fcvm.2021.641136 33718459 PMC7952317

[18] Zhou X Dudley SC Jr. Evidence for inflammation as a driver of atrial fibrillation. Front Cardiovasc Med. (2020) 7:62. 10.3389/fcvm.2020.00062 32411723 PMC7201086

[19] Scott L Jr Li N Dobrev D. Role of inflammatory signaling in atrial fibrillation. Int J Cardiol. (2019) 287:195–200. 10.1016/j.ijcard.2018.10.020 30316645 PMC6447485

[20] Korantzopoulos P Letsas KP Tse G Fragakis N Goudis CA Liu T. Inflammation and atrial fibrillation: a comprehensive review. J Arrhythm. (2018) 34:394–401. 10.1002/joa3.12077 30167010 PMC6111477

[21] Staerk L Preis SR Lin H Lubitz SA Ellinor PT Levy D. Protein biomarkers and risk of atrial fibrillation: the FHS. Circ Arrhythm Electrophysiol. (2020) 13:e007607. 10.1161/CIRCEP.119.007607 31941368 PMC7031024

[22] Yamagishi S-I. Role of advanced glycation endproduct (AGE)-receptor for advanced glycation endproduct (RAGE) axis in cardiovascular disease and its therapeutic intervention. Circ J. (2019) 83:1822–28. 10.1253/circj.CJ-19-0618 31366777

[23] Yamagishi SI Sotokawauchi A Matsui T. Pathological role of advanced glycation end products (ages) and their receptor axis in atrial fibrillation. Mini Rev Med Chem. (2019) 19:1040–48. 10.2174/1389557519666190311140737 30854960

[24] Berntsson J Smith JG Nilsson PM Hedblad B Melander O Engström G. Pro-atrial natriuretic peptide and prediction of atrial fibrillation and stroke: the malmö preventive project. Eur J Prev Cardiol. (2017) 24:788–95. 10.1177/2047487317693948 28195503

[25] Lubbers ER Murphy NP Mohler PJ. Defining the links between oxidative stress-based biomarkers and postoperative atrial fibrillation. J Am Heart Assoc. (2015) 4:e002110. 10.1161/JAHA.115.002110 25994438 PMC4599434

[26] Audebrand A Désaubry L Nebigil CG. Targeting GPCRs against cardiotoxicity induced by anticancer treatments. Front Cardiovasc Med. (2019) 6:194. 10.3389/fcvm.2019.00194 32039239 PMC6993588

[27] Böhm A Urban L Tothova L Bezak B Uher T Musil P. Concentration of apelin inversely correlates with atrial fibrillation burden. Bratisl Lek Listy. (2021) 122:165–71. 10.4149/BLL_2021_026 33618523

[28] Uher T Bohm A Urban L Tothova L Bacharova L Musil P. Association of apelin and AF in patients with implanted loop recorders undergoing catheter ablation. Bratisl Lek Listy. (2020) 121:484–7. 10.4149/BLL_2020_079 32990001

[29] Wang YZ Fan J Zhong B Xu Q. Apelin: a novel prognostic predictor for atrial fibrillation recurrence after pulmonary vein isolation. Medicine. (2018) 97:e12580. 10.1097/MD.0000000000012580 30278567 PMC6181607

[30] Folino A Montarolo PG Samaja M Rastaldo R. Effects of apelin on the cardiovascular system. Heart Fail Rev. (2015) 20:505–18. 10.1007/s10741-015-9475-x 25652330

[31] Cheng CC Weerateerangkul P Lu YY Chen YC Lin YK Chen SA. Apelin regulates the electrophysiological characteristics of atrial myocytes. Eur J Clin Invest. (2013) 43:34–40. 10.1111/eci.12012 23106642

[32] Casaclang-Verzosa G Gersh BJ Tsang TS. Structural and functional remodeling of the left atrium: clinical and therapeutic implications for atrial fibrillation. J Am Coll Cardiol. (2008) 51:1–11. 10.1016/j.jacc.2007.09.026 18174029

[33] Almuwaqqat Z O'Neal WT Norby FL Lutsey PL Selvin E Soliman EZ. Joint associations of obesity and NT-proBNP with the incidence of atrial fibrillation in the ARIC study. J Am Heart Assoc. (2019) 8:e013294. 10.1161/JAHA.119.013294 31564186 PMC6806039

[34] Büttner P Schumacher K Dinov B Zeynalova S Sommer P Bollmann A. Role of NT-proANP and NT-proBNP in patients with atrial fibrillation: association with atrial fibrillation progression phenotypes. Heart Rhythm. (2018) 15:1132–37. 10.1016/j.hrthm.2018.03.021 29604419

[35] Svennberg E Lindahl B Berglund L Eggers KM Venge P Zethelius B. NT-proBNP is a powerful predictor for incident atrial fibrillation - validation of a multimarker approach. Int J Cardiol. (2016) 223:74–81. 10.1016/j.ijcard.2016.08.001 27541645

[36] Izadi MR Ghardashi Afousi A Asvadi Fard M Babaee Bigi MA. High-intensity interval training lowers blood pressure and improves apelin and NOx plasma levels in older treated hypertensive individuals. J Physiol Biochem. (2018) 74:47–55. 10.1007/s13105-017-0602-0 29214526

[37] Fujie S Sato K Miyamoto-Mikami E Hasegawa N Fujita S Sanada K. Reduction of arterial stiffness by exercise training is associated with increasing plasma apelin level in middle-aged and older adults. PLoS ONE. (2014) 9:e93545. 10.1371/journal.pone.0093545 24691252 PMC3972107

[38] Yuzbashian E Zarkesh M Asghari G Hedayati M Safarian M Mirmiran P. Is apelin gene expression and concentration affected by dietary intakes? a systematic review. Crit Rev Food Sci Nutr. (2018) 58:680–8. 10.1080/10408398.2016.1262325 28125271

[39] Zuo H Svingen GFT Tell GS Ueland PM Vollset SE Pedersen ER. Plasma concentrations and dietary intakes of choline and betaine in association with atrial fibrillation risk: results from 3 prospective cohorts with different health profiles. J Am Heart Assoc. (2018) 7:e008190. 10.1161/JAHA.117.008190 29650710 PMC6015426

[40] Bertrand C Pignalosa A Wanecq E Rancoule C Batut A Deleruyelle S. Effects of dietary eicosapentaenoic acid (EPA) supplementation in high-fat fed mice on lipid metabolism and apelin/APJ system in skeletal muscle. PLoS ONE. (2013) 8:e78874. 10.1371/journal.pone.0078874 24244380 PMC3820669

[41] Kleinz MJ Davenport AP. Emerging roles of apelin in biology and medicine. Pharmacol Ther. (2005) 107:198–211. 10.1016/j.pharmthera.2005.04.001 15907343

[42] Kawamata Y Habata Y Fukusumi S Hosoya M Fujii R Hinuma S. Molecular properties of apelin: tissue distribution and receptor binding. Biochim Biophys Acta. (2001) 1538:162–71. 10.1016/S0167-4889(00)00143-9 11336787

[43] Castan-Laurell I Dray C Attané C Duparc T Knauf C Valet P. Apelin, diabetes, and obesity. Endocrine. (2011) 40:1–9. 10.1007/s12020-011-9507-9 21725702

[44] Japp AG Cruden NL Barnes G van Gemeren N Mathews J Adamson J. Acute cardiovascular effects of apelin in humans: potential role in patients with chronic heart failure. Circulation. (2010) 121:1818–27. 10.1161/CIRCULATIONAHA.109.911339 20385929

[45] Sepehri Shamloo A Bollmann A Dagres N Hindricks G Arya A. Natriuretic peptides: biomarkers for atrial fibrillation management. Clin Res Cardiol. (2020) 109:957–66. 10.1007/s00392-020-01608-x 32002634

[46] O'Neal WT Venkatesh S Broughton ST Griffin WF Soliman EZ. Biomarkers and the prediction of atrial fibrillation: state of the art. Vasc Health Risk Manag. (2016) 12:297–303. 10.2147/VHRM.S75537 27486329 PMC4957677

[47] Chong KS Gardner RS Morton JJ Ashley EA McDonagh TA. Plasma concentrations of the novel peptide apelin are decreased in patients with chronic heart failure. Eur J Heart Fail. (2006) 8:355–60. 10.1016/j.ejheart.2005.10.007 16464638

[48] Földes G Horkay F Szokodi I Vuolteenaho O Ilves M Lindstedt KA. Circulating and cardiac levels of apelin, the novel ligand of the orphan receptor APJ, in patients with heart failure. Biochem Biophys Res Commun. (2003) 308:480–5. 10.1016/S0006-291X(03)01424-4 12914775

[49] Scimia MC Hurtado C Ray S Metzler S Wei K Wang J. APJ acts as a dual receptor in cardiac hypertrophy. Nature. (2012) 488:394–8. 10.1038/nature11263 22810587 PMC3422434

[50] Kim YM Lakin R Zhang H Liu J Sachedina A Singh M. Apelin increases atrial conduction velocity, refractoriness, and prevents inducibility of atrial fibrillation. JCI Insight. (2020) 5:e126525. 10.1172/jci.insight.126525 32879139 PMC7526452

[51] Chamberland C Barajas-Martinez H Haufe V Fecteau MH Delabre JF Burashnikov A. Modulation of canine cardiac sodium current by apelin. J Mol Cell Cardiol. (2010) 48:694–701. 10.1016/j.yjmcc.2009.12.011 20036246 PMC2837777

