# Supplementary material for: Association Between Apelin and Atrial Fibrillation in Patients With High Risk of Ischemic Stroke
Source: Front Cardiovasc Med. 2021 Oct 12;8:742601. doi: 10.3389/fcvm.2021.742601 (PMC8545982; doi:10.3389/fcvm.2021.742601)
Supplement: Supplementary file 1 [file Data_Sheet_1.docx]

SUPPLEMENTARY MATERIAL

Association between apelin and atrial fibrillation in patients with high risk of ischemic stroke

Allan Bohm, MD, PhD, FESC, Peter Snopek, MD, Lubomira Tothova, PhD, Ass. Prof., Branislav Bezak, MD, Nikola Jajcay, PhD, Marianna Vachalcova MD, PhD,

Tomas Uher MD, Marian Kurecko MD, PhD, Viera Kissova MD, PhD, MPH,

Katarina Danova MD, PhD, Peter Olejnik MD, PhD, Ass. Prof., Peter Michalek MD, PhD, Tereza Hlavata MD, Katarina Petrikova, MSc, Viliam Mojto MD, PhD, Ass. Prof., MPH, MHA, Jan Kyselovic Prof, PharmDr, PhD, Stefan Farsky, MD, PhD, Ass. Prof., FESC

Correspondence to: allan.bohm@gmail.com

## **Supplemental Results**

*Statistical analysis*

In order to determine the differences in apelin concentrations between the three groups, we employed analysis of variance (ANOVA) test. A test for normality yielded one group (patients without atrial fibrillation) as not normal (*W=0.926, p=0.039*), but subsequent analysis using Q-Q plots against normal distribution showed the deviation from normal distribution as minimal (Supplementary Fig. 1). Therefore, we opted to use parametric analyses over non-parametric since they offer more statistical power. As all three groups have equal variances (*W=0.58, p=0.944*), we resorted to classical one-way ANOVA. ANOVA indicated significant group effect on apelin concentrations with *F(2, 90)=10.67, p<0.001, η_p_^2^=0.192* with statistical power *0.994,* given our number of participants. Subsequent analysis uncovered, that the difference in apelin concentration between healthy controls and patients with atrial fibrillation (AF) was significant (*0.982 ± 0.060* ng/ml vs. *0.694±0.148* ng/ml, *p=0.001, d=1.044*) as well as between patients with and without AF (*0.694±0.148* ng/ml vs. *0.975±0.458* ng/ml, *p=0.001, d=-1.021*), respectively. The difference between healthy controls and patients without AF was not significant (*0.982±0.060* ng/ml vs. *0.975±0.458* ng/ml, *p=0.900, d=0.023*)  (Supplementary Fig. 2).

After establishing significant differences in apelin concentrations within our dataset, we correlated apelin concentrations with diastolic dysfunction scale (*Spearman’s r=-0.126, CI95% [-0.37, 0.13], p=0.341*), left atrium diameter in parasternal short axis [mm] (*Spearman’s r=-0.097, CI95% [-0.34, 0.16], p=0.466*) and NT-proBNP [ng/l] (*Spearman’s r=-0.147, CI95% [-0.39, 0.11], p=0.267*). As seen from the correlation and p-values, none of them were deemed significant (Supplementary Fig. 3).

Given the significant differences of apelin concentrations, we assumed that apelin would be a good predictor of AF. To this end, we computed a receiver operating characteristic (ROC) of apelin as an AF predictor using only patient’s data in order to have balanced classes (AF vs. no AF). The full curve can be seen in Supplementary Figure 4 and the area under the curve (AUC) of apelin predictor reached *0.658*.

The ideal threshold in this dummy classifier was determined as a threshold that maximizes *true positive rate – false positive rate*variable (*Youden index*), and was computed as *apelin [ng/ml] = 0.969* (hence all patients with apelin lower that this threshold, were classified as AF). By setting this threshold, we obtained classification accuracy of *0.712*, sensitivity of *0.966*, and specificity of *0.467*.

Finally, we built a logistic regression model for classifying AF using multiple predictors, including apelin. We compared two approaches to this problem, with the first being the manual feature selection based known predictors of AF from available literature. We selected 16 predictors from our gathered data and fitted a logistic regression model using our patients’ data. The model trained on all data scored *AUC = 0.875*(Supplementary Fig. 5).

The full list of predictors with their coefficients and p-values can be seen in Supplementary Table 1. Only 2 predictors were statistically significant with p-values lower than 0.05: *apelin*, and *NT-proBNP*.

The second, data-driven, route was to compute bivariate analysis (significant differences in our dataset between AF and no AF patients) and include all predictors, whose differences between groups had p-value lower 0.1 (based on t-test, Mann-Whitney U-test, or χ^2^ test where appropriate). Differences in medication were not included in this analysis because they directly depend on the presence of AF.

This landed us with 4 predictors (of course, including apelin) and the final model scored *AUC=0.825* (Supplementary Fig. 6). In this model, only apelin scored p-value lower than preselected threshold of 0.05. *The full list of predictors with their coefficients and p-values can be seen in Supplementary Table 2.* Since in the above logistic regression we fitted the model with all our data in order to see how different predictors help to classify AF, we cannot really see the performance of the model on unseen data. In order to assess the true model performance, we selected predictors from our bivariate analysis with p-value < 0.1, and repeatedly trained logistic regression model using repeated stratified K-Fold cross-validation strategy. In stratified K-Fold, the model is trained and validated *k* times, when in each round the dataset was divided into training and testing data based on number of folds. The receiver operating characteristic (ROC) was computed only from testing dataset. The model fitting was done using 5 folds (hence 20% of the data is reserved for testing) in 20 repeats such that we have a good overall statistic. Our final model scored *AUC = 0.725 ± 0.131*, and the full ROC curve showed as mean *±* one standard deviation can be seen in Supplementary Figure 7.

## **Supplementary Figures**


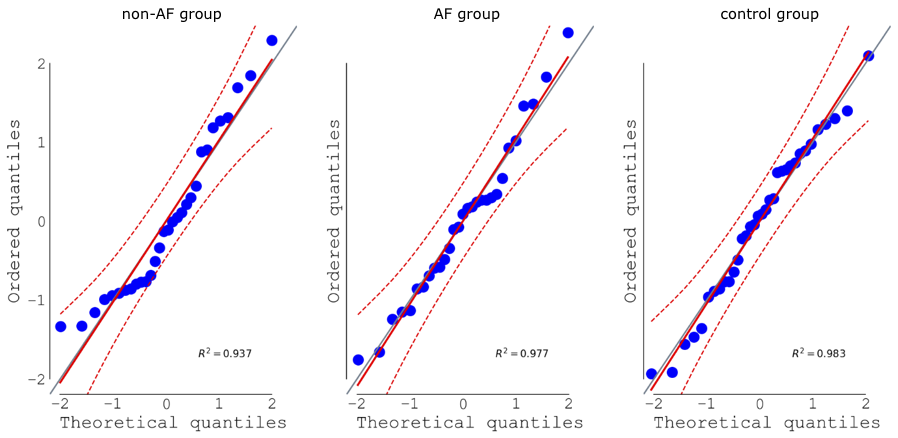


**Supplementary Figure 1:** Normality test: Q-Q plots for non-atrial fibrillation patients (non-AF group), atrial fibrillation patients (AF group) and control group.


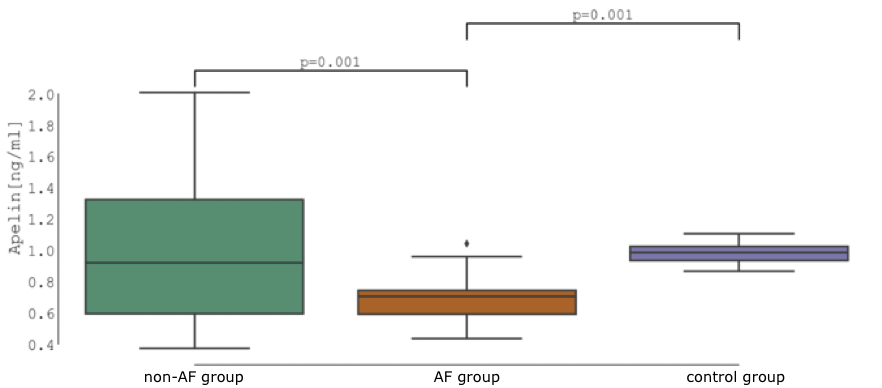


**Supplementary Figure 2:** Apelin concentration: Non-atrial fibrillation patients (non-AF group) vs. atrial fibrillation patients (AF-group) vs. control group.


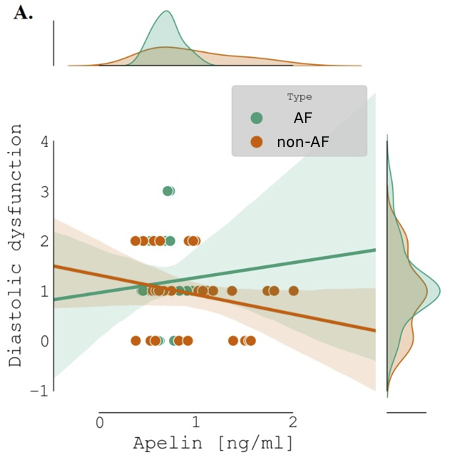

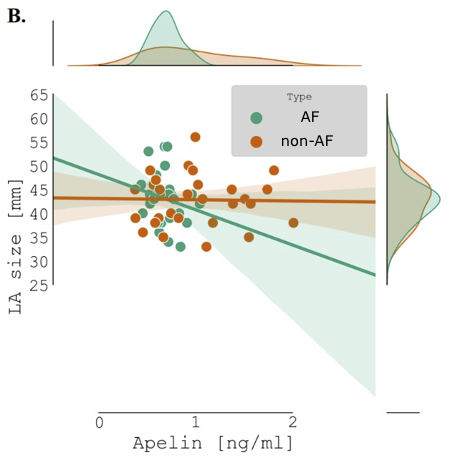

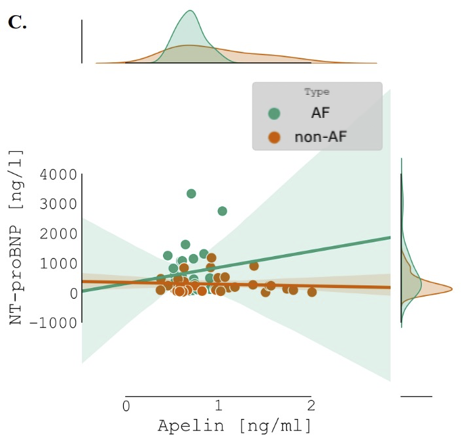


**Supplementary Figure 3:** Apelin concentration: Non-atrial fibrillation patients (no-AF) vs. atrial fibrillation patients (AF): (A) correlation between apelin concentration and diastolic dysfunction; (B) correlation between apelin concentration and left atrium diameter; (C) correlation between apelin concentration and NT-proBNP.


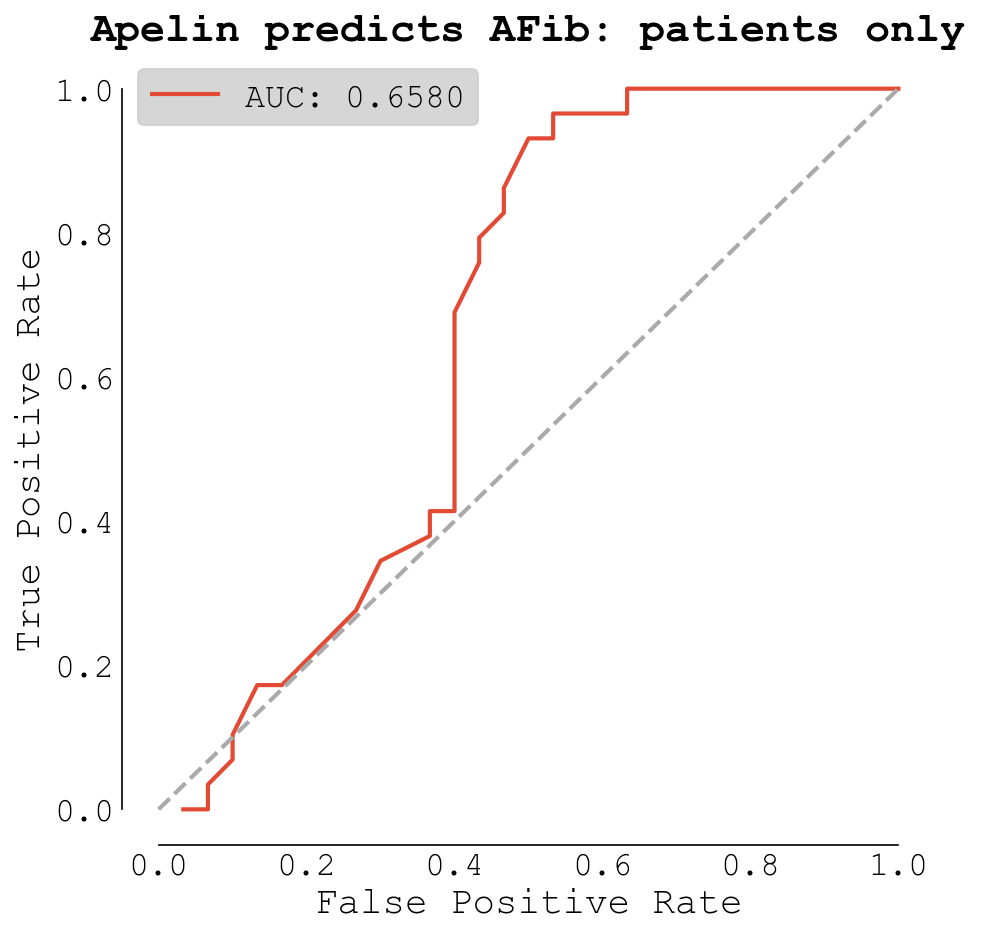


**Supplementary Figure 4:** Receiver operating characteristic (ROC) analysis of apelin as a predictor of atrial fibrillation (AF).


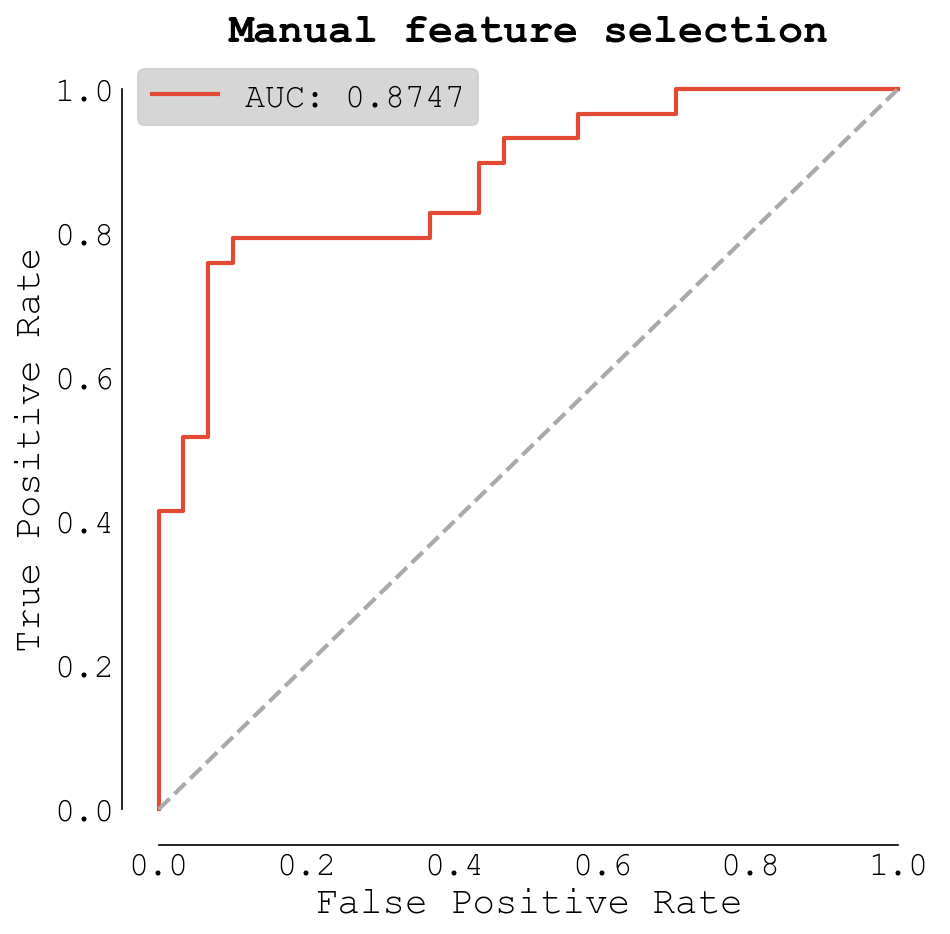


**Supplementary Figure 5:** Logistic regression model (all patient data).


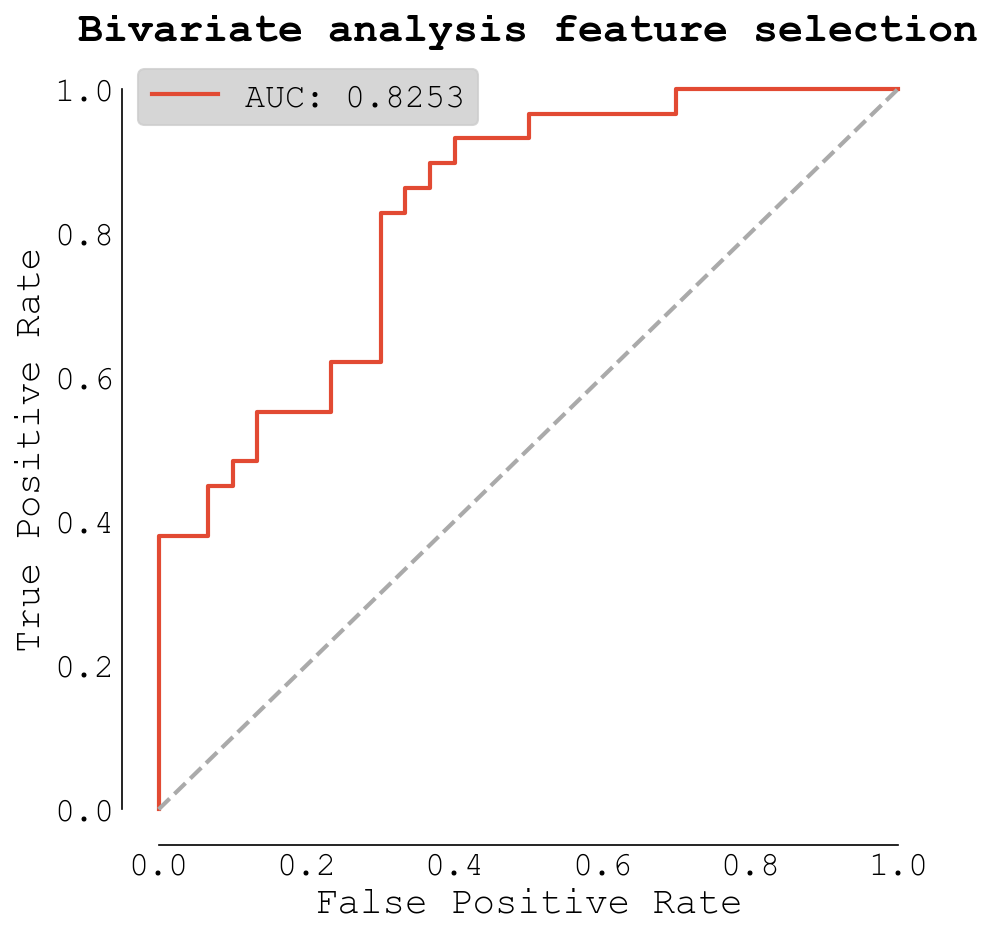


**Supplementary Figure 6:** Logistic regression model based on selection from bivariate analysis (predictors with p < 0.1).


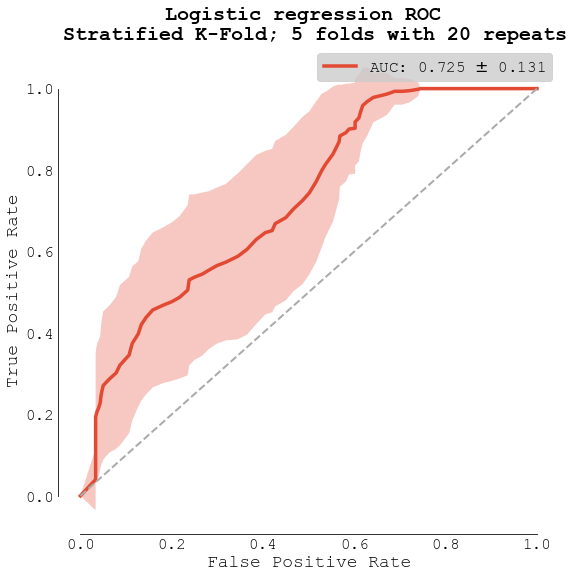


**Supplementary Figure 7:** Logistic regression model using repeated stratified K-Fold cross-validation strategy.

**Supplementary Tables**

**Supplementary Table 1.** Logistic regression model for AF predictors.

| **Predictor** | **Coef** | **(95% CI)** | **p-value** |
| --- | --- | --- | --- |
| **(Intercept)** | -2.875 | (-5.958 to  0.209) | 0.068 |
| **Signs of heart failure (%)** | -0.606 | (-2.856 to 1.645) | 0.598 |
| **Diastolic dysfunction (Grade)** | 1.512 | (-0.622 to 3.647) | 0.165 |
| **Chronic obstructive pulmonary disease (COPD) (%)** | 3.745 | (0.005 to 7.485) | 0.05 |
| **Vascular disease (%)** | -0.175 | (-2.005 to 1.656) | 0.852 |
| **Gender (%)** | 1.64 | (-0.495 to 3.775) | 0.132 |
| **Diabetes Mellitus (%)** | 1.252 | (-1.073 to 3.577) | 0.291 |
| **D-Dimer (ug/ml)** | -0.325 | (-1.19 to 0.541) | 0.462 |
| **Systolic blood pressure (mmHg)** | 0.263 | (-0.701 to 1.228) | 0.593 |
| **Age (years)** | -0.47 | (-1.398 to 0.457) | 0.320 |
| **NT-proBNP (ng/l)** | 1.823 | (0.251 to 3.396) | 0.023 |
| **Diastolic blood pressure (mmHg)** | -0.45 | (-1.447 to 0.548) | 0.377 |
| **BMI (kg/m2)** | 0.047 | (-0.916 to 1.011) | 0.923 |
| **Apelin (ng/ml)** | -1.936 | (-3.551 to -0.320) | 0.019 |
| **CRP (mg/l)** | -0.222 | (-1.068 to 0.624) | 0.607 |
| **Creatinine (umol/l)** | 0.464 | (-0.605 to 1.534) | 0.395 |
| **Diameter of left atrium in PLAX (Parasternal long axis) (mm)** | -0.645 | (-1.81 to 0.520) | 0.278 |

Abbreviations: AF, atrial fibrillation; BMI, body mass index; CRP, C-reactive protein; NT-proBNP, N-terminal fragment of brain natriuretic peptide

**Supplementary Table 2.** Logistic regression model for AF predictors based on selection from bivariate analysis (predictors with p < 0.1).

| **Predictor** | **Coef** | **(95% CI)** | **p-value** |
| --- | --- | --- | --- |
| **(Intercept)** | 0.409 | (-0.653 to 1.471) | 0.450 |
| **Apelin (ng/ml)** | -1.0190 | (-1.915 to -0.123) | 0.026 |
| **Hs-troponin (ng/l)** | 3.907 | (-1.512 to 9.327) | 0.158 |
| **NT-proBNP (ng/l)** | 0.777 | (-0.142 to 1.696) | 0.097 |
| **CRP (mg/l)** | -0.321 | (-1.061 to 0.418) | 0.395 |

Abbreviations: AF, atrial fibrillation; CRP, C-reactive protein; Hs-troponin, High-sensitivity troponin; NT-proBNP, N-terminal fragment of brain natriuretic peptide
